# Simple spatial scaling rules behind complex cities

**DOI:** 10.1038/s41467-017-01882-w

**Published:** 2017-11-28

**Authors:** Ruiqi Li, Lei Dong, Jiang Zhang, Xinran Wang, Wen-Xu Wang, Zengru Di, H. Eugene Stanley

**Affiliations:** 1 School of Systems Science, Beijing Normal University, 100875 Beijing, China; 2 Center for Polymer Studies and Physics Department, Boston University, Boston, MA 02215 USA; 3 School of Architecture, Tsinghua University, 100084 Beijing, China; 4 College of Resources Science and Technology, Beijing Normal University, 100875 Beijing, China

## Abstract

Although most of wealth and innovation have been the result of human interaction and cooperation, we are not yet able to quantitatively predict the spatial distributions of three main elements of cities: population, roads, and socioeconomic interactions. By a simple model mainly based on spatial attraction and matching growth mechanisms, we reveal that the spatial scaling rules of these three elements are in a consistent framework, which allows us to use any single observation to infer the others. All numerical and theoretical results are consistent with empirical data from ten representative cities. In addition, our model can also provide a general explanation of the origins of the universal super- and sub-linear aggregate scaling laws and accurately predict kilometre-level socioeconomic activity. Our work opens a new avenue for uncovering the evolution of cities in terms of the interplay among urban elements, and it has a broad range of applications.

## Abstract (web summary)

Cities can be treated as dynamic complex systems being controlled by the interactions among people, whilst the detail remains largely unknown. Li et al. use spatial attraction together with matching growth to unify population, roads, and socioeconomic interactions crossing ten representative cities.

## Introduction

Archaeological studies indicate that although the first Homo sapiens appeared ~200 thousand years ago^1^, the earliest cities emerged only 5–6 thousand years ago^2^. Today cities world-wide have attracted more than half of the population^3^, created at least 80% of the wealth^4^, and generated >90% of the innovation^5^. They also have brought such modern problems as crime, pollution, and traffic congestion^6, 7^. Cities are typical complex systems composed of different elements, such as buildings, people, roads, traffic, and interactions. The efforts to understand these elements have tended to examine them individually, focusing on, for example, city morphologies^3, 8–12^, population distributions^13–17^, area-population allometry^15, 16, 18^, road networks^19–22^, pollution and congestion^23–25^, wealth and innovation creation^26–28^, and rent prices^29, 30^. However, cities are not simple combination of independent pieces but integration of many facets, including infrastructural, economic, and social elements^3^. Thus if we are to quantitatively describe their features, we must approach cities as interactive, interdependent complex systems, and not treat each city element separately.

Over the past decade, considerable progress has been made in study of scaling laws by revealing significant role of populations in determining and predicting output, innovation, crime, road volume, and other aggregate city variables^26, 27, 31, 32^. The scaling variables fall into three universality classes according to their functionality: interaction-related variables with super-linear scalings, infrastructural variables with sub-linear scalings, and household-related variables with nearly linear scalings. Note that hierarchical structure^33^ gives rise to the scaling laws and accounts for their connections and correlations. The scaling theory of cities provides quantitative evidence for the economies of scale that underpin the sustainable development of cities^3^. Other studies focus on the spatial distributions of different elements, such as built-up areas^11^, population^13, 14, 16^, road networks^20, 21^, and socioeconomic interactions^34^ within a city.

Despite advances in the study of cities, we still lack a unified theoretical framework for the spatial distributions of various elements within cities and macroscopic scaling laws across cities. Both the interplay among elements at the microscopic level and the relationship between spatial distributions and aggregate scaling laws remain outstanding problems. Addressing them is important to urban planning^17^, traffic engineering^35^ and transportation efficiency^36^, infectious disease epidemiology^37, 38^, and emergency management^39^, among other applications.

In this paper, we use a dynamic model to interpret the evolution of both spatial distributions and aggregate scaling laws. Specifically, we propose a simple spatial attraction (SA) mechanism that combines competition between population aggregation and exploration of new areas in a city to determine the spatial distribution of an active population (AP). AP is a mixture of residential and working populations^10, 40^ according to the duration of their activities in the region. This is a more appropriate proxy than simply residential population for estimating socioeconomic activities. The demand for socioeconomic interactions among AP agglomerations drives the growth and expansion of road networks. The development of road networks facilitates socioeconomic interactions among different regions. Thus a local AP plays a deterministic role in the evolution of city populations, road networks, and socioeconomic interactions. Our model successfully reproduces the spatial distributions of them and reveals that their spatial scalings of the distance from the central area are in a consistent form that allows us to obtain other spatial scalings from a single observation. In addition, such spatial scalings explain the origins of the macroscopic laws associated with both super- and sub-linear scalings of aggregate variables. And our theoretical analysis is based on dynamic growth not static assumptions, and this goes beyond the methods used in previous researches^26, 33, 41^. Our model is also able to use the local AP to accurately predict the local density of such elements as kilometre-level socioeconomic interactions in Greater London as indicated by nighttime light luminosity intensities.

## Results

### A unified model based on a spatial attraction and matching growth mechanism

**Fig. 1:**
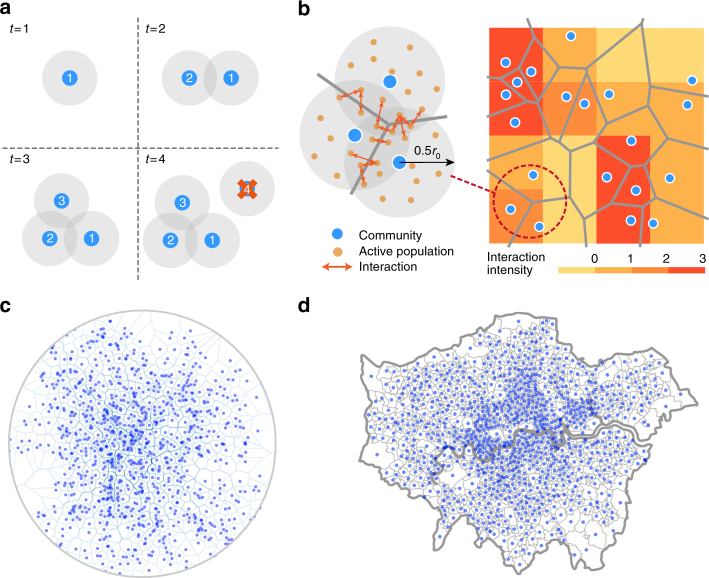
Illustration of the model and simulated AP distribution by the model and empirical data. **a** A sketch of the spatial attraction process. The numbers in nodes are the orders with which they join the system. The large grey disks with radius *r*_0_/2 around the nodes are their ranges of interconnections. A node can survive only if its grey disk overlaps with the existing disks of other nodes (i.e., the entire grey shaded area). Therefore, node 4 cannot survive. **b** The road network generated by a Voronoi tessellation based on the AP distribution and the corresponding socioeconomic interaction distribution calculated according to the rules we assumed. The colours in grids represent local interaction intensity. (Inset: the micro-socioeconomic interaction pattern of AP). **c** Simulated AP distribution. **d** Empirical population distribution of London. A node represents an MSOA (see Methods) in Greater London, which possesses a population of approximately 5000

Assume an *L* × *L* (*L* → ∞) lattice in a 2-D Euclidean space where the agglomeration occurs and the city grows. The basic agglomeration unit is an active community with a constant AP, which is a node within the lattice. Here the AP is not individual people but interactive units in a given place. Initially we generate an active community (i.e., the seed node) at the centre of the lattice (see Methods for more discussions about the initial situations with multiple seed nodes and adding a new seed node after a certain number of time steps). Inspired by ref.^42^, we come up with a SA mechanism which assumes that at each time step a new active community is born at a random position (*r*, *θ*) with a probability

1 $${\prod} {(r,\theta ,t) \propto \rho (r,\theta ,t) + C} ,$$

where *r* is the distance to the central area (i.e., the position of seed node (0, 0)), *θ* is the polar angle of the new node, and *ρ*(*r*, *θ*, *t*) is the local density of the AP at location (*r*, *θ*) and time *t*. *C* is a free parameter characterising the attraction of an empty place for habitation or development, which can be a proxy of attraction for natural endowment or policy effects on land constraints. The new node survives if it is sufficiently close to an existing node (i.e., the Euclidean distance is less than a given threshold *r*_0_). If not, it will fail to join the city (see Fig. 1a) due to too high cost for making connections. This is what we called matching growth. In our model, *r*_0_ is a constant which can be determined by the technological and transportation development level.

The SA mechanism quantifies the competition between population aggregation (the first term in Eq. (1) and the survival rule) and the search for free space (the second term in Eq. (1)) for a newcomer to the city. *C* is the parameter measuring the relative effects of the two factors on city evolution. Parameter *C* denotes the natural attraction of a place in the absence of any population. When *C* = 0, all communities aggregate in the central area. For large values of *C*, communities disperse across the city.

### Construction of road networks based on AP

By our one-parameter model we can qualitatively reproduce the AP agglomeration patterns (see Fig. 1c). As the scale of this population aggregation becomes larger, the demand for interactions among people drives the growth and expansion of road networks. We assume that the typical length of a road segment *l*_0_ between two nodes depends on the local AP density in the form *ρ* ^−1/2^, and the total length *l*(*r*, *θ*, *t*) per capita area in an infinitesimal space at location (*r*, *θ*) at time *t* is

2 $$l(r,\theta ,t) \propto \rho (r,\theta ,t)^{1/2},$$

which is supported by dimensional analysis and empirical studies^19, 20^. Several representative models can yield these relationships. For example, in the simplest situation, and for strict fairness (see Supplementary Note 2), we can assume that road networks are Voronoi polygons around the nodes (all simulations in this paper adopt this rule). Other road network generating approaches, such as a minimum spanning tree (see Supplementary Note 2 and Supplementary Figs. 4–6) or the road network model in ref.^20^ also yields Eq. (2).

### Socioeconomic interactions along roads

The agglomeration of people and the construction of road networks facilitate the socioeconomic interactions essential to economic output and innovation. We assume that all interactions occur along roads^33^, and that the socioeconomic output (e.g., gross regional﻿ demostic product, GRDP) of a city is approximately proportional to the total number of socioeconomic interactions^28, 33^. This is in accordance with the observation that all markets, companies, and shops are built along roadsides. Thus together with Eq. (2), the relationship between the strength of socioeconomic interactions *g*(*r*, *θ*, *t*) and the AP density *ρ*(*r*, *θ*, *t*) in the infinitesimal space is

3 $$g(r,\theta ,t) \propto \rho (r,\theta ,t)l(r,\theta ,t) \propto \rho (r,\theta ,t)^{3/2}.$$

### Spatial scaling

Our simple rules produce the AP, road network, and socioeconomic interaction distributions (see Fig. 1b, c). The AP distribution and its morphology are in good agreement with the real city of Greater London (see Fig. 1d).

**Fig. 2:**
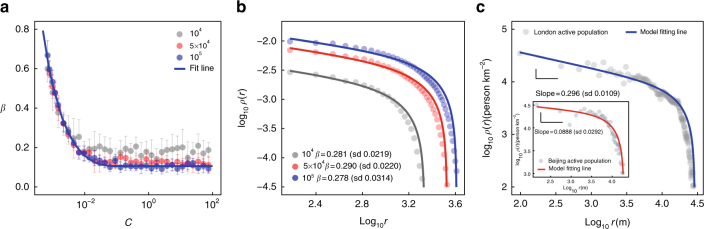
Population density distribution by the model and validation with empirical data. **a** The relationship between *C* and *β* for different sizes (*P*(*t*) = 10^4^, 5 × 10^4^, 10^5^), which can be well fitted by a power law with an exponential cutoff ($\beta \sim 0.00350C^{ - 0.697}e^{ - 46.280C}, R^2 = 0.996$, see Supplementary Fig. 1 and Supplementary Note 1 for more details). *β* is estimated by the non-linear least squares (NLS) fitting of the simulation. Error bars mean±sd, obtained from ten different realisations. **b** Simulated AP density on a log-log plot along the distance for various system sizes with *C* = 0.002 and *P*(*t*) = 10^4^, 5 × 10^4^, 10^5^. The absolute density increases as the system grows larger (see Supplementary Fig. 2 and Supplementary Note 1 for the re-scaled population density). **c** The empirical AP density along the distance to the central areas of London (*β* = 0.296) and Beijing (Inset, *β* = 0.0888) (see Table S1 for further details on the central areas of cities)

**Fig. 3:**
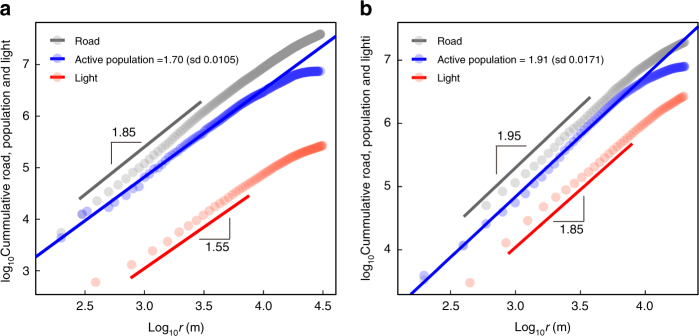
The spatial scaling sresults for two cities. Results for **a** London and **b** Beijing. The exponents of AP in London and Beijing are 1.70 and 1.91, respectively, which are estimated by linear regression in **a**, **b**. Then, *β* = 0.30 for London and 0.09 for Beijing can be estimated according to Eq. (5). According to our theory, the exponents of road length and socioeconomic interactions captured by nighttime lights can be predicted to be 2 − *β*/2 = 1.85(1.95) and 2 − 3*β*/2 = 1.55(1.86) for London (Beijing), which are very close to the empirical results—by linear regression, the exponents and standard deviations for road length and socioeconomic interactions of London are 1.85 (sd 0.00800) and 1.66 (sd 0.0118). For Beijing, they are 1.95 (sd 0.0164) and 1.74 (sd 0.0105), respectively

The model allows us to quantify these predictions. According to the SA mechanism, when *t* is sufficiently large the spatial distribution of AP is approximately

4 $$\rho (r,t) \propto r^{ - \beta }\left( {R(t)^{1 + \beta } - r^{1 + \beta }} \right)\sim r^{ - \beta },$$

where *ρ*(*r*, *t*) is the average density at distance *r* from the city centre, i.e., the seed node at position (0, 0), *R*(*t*) is the radius of the entire city at time *t* (see Fig. 2b and further details of the explicit form of *ρ*(*r*, *t*) are in Supplementary Note 1), and *β* is a parameter derived from *C*. The dependence of *β* on *C* (see Fig. 2a) can be derived by the non-linear fitting, and the simulation results are shown in Fig. 2b. Figure 2c shows that the collected data validate Eq. (4). The AP in the real data is defined as a mixture of working and residential populations according to their active duration in that region (see Methods). In the downtown area (i.e., when *r*/*R*(*t*) → 0), the AP density decays following the power law shown in Eq. (4) (see Fig. 2c). In contrast, residential population density decays exponentially as we move from city centre to urban fringe, as verified in previous research^13^ and our data (see Supplementary Fig. 8). This reconciles the conflict between area-size allometry and the exponential decay of population from city centre to urban fringe found in the literature^16^. To eliminate the influence of noise in the empirical data and allow an unbiased comparison of different quantities, we study the cumulative quantities in terms of the distance from the central area instead of the local density (see Fig. 3). Integrating Eq. (4) we obtain

5 $$\begin{array}{*{20}{l}} {P(r,t)} \hfill & \hskip-8pt = \hfill &\hskip-7pt {{\int}_0^r {\int}_0^{2\pi } \rho (x,\theta ,t){\it x}\mathrm{d}{\it x}\mathrm{d}\theta } \hfill \\ {} \hfill & \propto \hfill & {r^{2 - \beta }\left( {\frac{{R(t)^{1 + \beta }}}{{2 - \beta }} - \frac{{r^{1 + \beta }}}{3}} \right)\sim r^{2 - \beta },} \hfill \end{array}$$

where *P*(*r, t*) is the cumulative AP within the concentric circle of the central area with radius *r* (see Supplementary Note 3 and Supplementary Fig. 7 for detailed derivations). This expression is approximately a power law with an exponent 2 − *β* when $r \ll R(t)$. This is consistent with the fractal city hypothesis^11^, and the fractal dimension is 2 − *β* if the distributions of APs and buildings are similar. We can similarly calculate the cumulative road length and socioeconomic interactions as

6 $$L(r,t) = {\int}_0^r {\int}_0^{2\pi } l(x,\theta ,t)x\mathrm{d}x\mathrm{d}\theta \sim r^{2 - \beta /2},$$

and

7 $$G(r,t) = {\int}_0^r {\int}_0^{2\pi } g(x,\theta ,t){\it x}\mathrm{d}{\it x}\mathrm{d}\theta \sim r^{2 - 3\beta /2}.$$

Therefore the exponents of the cumulative length of the road network and socioeconomic interactions within the circle of radius *r* are 2 − *β*/2 and 2 − 3*β*/2. These predictions are consistent with the data from Beijing and London shown in Fig. 3. Due to the need for high-resolution data, we use nighttime light as the proxy for socioeconomic interactions^43–45^. Although nighttime light data has some intrinsic disadvantages when representing high-resolution interactions (e.g., at a community level, because luminosity is also associated with road density and type of land use), it remains the best candidate among open-source data. We show that as long as the spatial resolution is not excessively high it is a good proxy (see Methods, Supplementary Figs. 10 and 12–14 and Supplementary Note 6 for further discussion). Thus based on Eqs. (5)–(7) we can obtain all the other spatial scaling exponents from any single observation.

### Aggregate scaling laws

In addition to providing the spatial distributions of the three main variables, the model is able to generate the scaling laws at the aggregate level, i.e., the dependencies of total area, road length, and GDP on population. Note that Eqs. (2) and (3) are power functions and thus we can use the following general form to facilitate our discussion

8 $$y \propto \rho ^\eta ,$$

where *y* can represent *ρ*, *l*, or *g*, and the corresponding *η* = 1, 1/2, or 3/2, respectively. If we take *η* = 0, this enables us to calculate the total area of the city in Eq. (9). Using the spatial scaling of the AP shown in Eq. (4), we can measure how the aggregate variables are dependent on the population by integrating the densities of both sides to the upper limit *R*(*t*)

9 $$Y(t) = {\int}_0^{R(t)} {\int}_0^{2\pi } \rho ^\eta (r,\theta ,t)r\mathrm{d}{\it r}\mathrm{d}\theta \sim R({\it t})^{2 + \eta },$$

where *Y*(*t*) is the gross quantities of area *A*(*t*), population *P*(*t*), total road length *L*(*t*), and total number of socioeconomic interactions *G*(*t*) when *η* = 0, 1, 1/2, and 3/2 according to Eqs. (5)–(7), respectively. We also test these relations using data from the U.S. cities, which give us similar exponents. The model predictions for *P*(*t*), *L*(*t*), and *G*(*t*) are 3.0, 2.5, and 3.5, and the empirical results are 2.87, 2.40, and 3.23, respectively (see Supplementary Note 3 and Supplementary Figs. 12 and 13 for the details of empirical results). The small gap between the empirical results and the model predictions may be caused by the geographical and hydrological conditions of the city, which also partially explain why cities are fractal in form^11^. Thus the integration in Eq. (9) is not over a complete circular area with a radius *R*(*t*). Because these variables are all functions of the radius *R*(*t*) of a city, we can obtain all the scaling laws for the city area, road length, and GDP using total population. Their exponents are 2/3, 5/6, and 7/6, respectively, and all are consistent with empirical data (see Table 1, and see Supplementary Note 3 for detailed derivations of scaling laws). Note that the scaling laws are independent of the exclusive parameter *β*, which is cancelled out in the calculation.

**Table 1:** Theoretical and empirical results for scaling laws

Variables	Theoretical	Empirical^a^
Road length	5/6	(0.74, 0.92)
GDP	7/6	(1.01, 1.33)
Area	2/3	(0.56, 1.04)^b^

^a^Data from ref.^33^

^b^Based on different definitions of city area, the exponents are quite controversial^25^

### Predictions

As our model has only one free parameter, we can estimate *β* using the empirical spatial scaling of any single element to predict the other elements. Figure 3 shows the use of AP to predict road length and socioeconomic interactions for London and Beijing. Table 2 shows the use of road length instead of AP to predict other spatial scalings (see Supplementary Table 1 for more details). In this case there is a lack of AP data for most cities. The data is either inaccessible, or limited by the statistical approach used (see Supplementary Note 5 for details).

**Table 2:** Exponents for the spatial distributions of variables for ten representative cities

City	Estimated Road^a^	Estimated *β* ^b^	Predicted Light^c^	Empirical Light^d^	Predicted AP^e^
Amsterdam	1.79 ± 0.02	0.42 ± 0.04	1.37 ± 0.06	1.61 ± 0.04	1.58 ± 0.04
Beijing	1.95 ± 0.03	0.10 ± 0.06	1.85 ± 0.09	1.74 ± 0.02	1.90 ± 0.06
Berlin	1.81 ± 0.01	0.38 ± 0.02	1.43 ± 0.03	1.68 ± 0.05	1.62 ± 0.02
Budapest	1.86 ± 0.02	0.28 ± 0.04	1.58 ± 0.06	1.70 ± 0.08	1.72 ± 0.04
Lille	1.85 ± 0.04	0.30 ± 0.08	1.55 ± 0.12	1.73 ± 0.06	1.70 ± 0.08
London	1.85 ± 0.02	0.30 ± 0.04	1.55 ± 0.06	1.66 ± 0.02	1.70 ± 0.04
Los Angeles	1.85 ± 0.01	0.30 ± 0.02	1.55 ± 0.03	1.61 ± 0.03	1.70 ± 0.02
Milan	1.87 ± 0.02	0.26 ± 0.04	1.61 ± 0.06	1.74 ± 0.05	1.74 ± 0.04
Prague	1.86 ± 0.01	0.28 ± 0.02	1.58 ± 0.03	1.75 ± 0.09	1.72 ± 0.02
Tokyo	1.81 ± 0.01	0.38 ± 0.02	1.43 ± 0.03	1.73 ± 0.04	1.62 ± 0.02

^a^Exponents of road length spatial scalings are estimated by ordinary least squares. The number after ± indicates 95% confidence interval

^b^Calculated *β*s according to the exponents in the second column based on Eq. (2)

^c^Predicted exponents of nighttime light spatial scalings according to 2 − 3*β*/2

^d^Estimated exponents of empirical nighttime light spatial scalings for different cities

^e^Predicted exponents of AP spatial scalings according to 2 − *β*

**Fig. 4:**
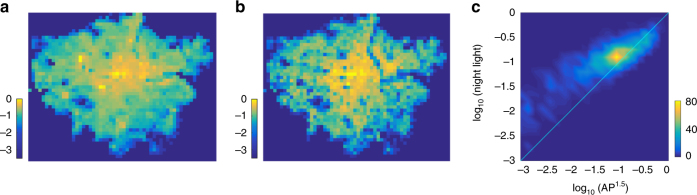
Prediction of kilometre-level socioeconomic activity. **a** The rasterised bit map of nighttime light from VIIRS in Greater London, which is pruned by the outliers of the AP distribution (we also set the value of a nighttime light pixel as 0 if there is no population within it). **b** The predicted spatial distribution of socioeconomic interactions according to *ρ* ^3/2^ (Eq. (3)). **c** Colour map of the scatter plot for correlation between nighttime light and AP 1.5 power. Brighter areas indicate that more data points fall in that small region and the colour bar indicates the number of it. The data are all normalised by the respective maximal value in **a**, **b**. Each pixel in **a**, **b** corresponds to a square with an edge length of 1 km

We can also make kilometre-level predictions when we know the AP in terms of the relationships between the densities of the relevant variables in Eqs. (2) and (3). Figure 4b, for example, shows predictions of local night-time light densities in Greater London at the kilometre level using the local AP densities according to Eq. (4). Compared with Fig. 4a, although there are larger deviations in the regions with low nighttime light intensity, the predictions remain accurate in most regions (see Fig. 4c and Supplementary Fig. 14).

## Discussion

In this paper, we have proposed a simple mechanism, namely spatial attraction together with matching growth, to simulate city growth and reveal the hidden spatial scaling relations between different city elements. The process reveals the competition between population aggregation and the exploration of empty space, which is controlled by a single parameter *C*. The model can yield all the spatial scalings of the three main elements (APs, road networks, and socioeconomic interactions), and the theoretical analysis is consistent with the empirical results. We found theoretically that the spatial parameter *β* derived from *C* plays a key role in all spatial scalings. Specifically, when *β* is large, all three elements concentrate in the downtown area. In contrast, a small *β* value indicates a dispersal of urban elements. We find that the *β* of Beijing is smaller than that of London, suggesting that Beijing is undergoing a more flat expansion than London. Thus the *β* value is an important characteristic of a city because it can capture both the static spatial patterns of the elements and the developmental status of the city. In addition, our model offers a precise prediction of local socioeconomic interactions, as reflected by nighttime light intensity in London, and this can be applied to many issues pertaining to city development.

Furthermore, our simple model can also produce the super-linear and sub-linear scaling laws of aggregate variables, including urban area, total road length, and GDP. The scaling exponents are unaffected by the parameter of the spatial scalings but strongly affected by the relationships between local element densities. Thus our model explains the emergence of the aggregate scaling laws that are determined by local interacting rules shown in Eqs. (2) and (3). So our model can both explain the mesoscopic spatial distribution and aggregate scaling laws, and provides new insights into both urban modelling and the quantitative theory of cities.

In addition, our theoretical analysis is based on dynamic growth not static configuration, and this goes beyond the methods used in previous researches^26, 33, 41^.

Beyond the three elements considered in this paper, we can also make predictions on other variables with proper rules. For example, following the assumption in ref.^33^, if we assume that the average rent price of a given city is proportional to the total output divided by its area, we can derive a scaling of a 0.5 power between rent price and population (see Supplementary Note 7). When we assume the local rent prices is proportional to demand and local economic development level, we find that the local rent price decays along the distance in a scaling form $p(r)\sim r^{ - 5/2\beta }$, which is supported by the empirical data for Beijing (see Supplementary Fig. 11 and Note 7).

Our work also raises some questions that, when answered, can further deepen our understanding of various city phenomena. As cities expand, two or more neighbouring cities will inevitably form large metropolitan areas, such as New York, Boston, and Yangtze Rive Delta region in China. Our current model assumes that a city grows in an ideal isotropic space, does not take into consideration real geographic and hydrological factors, and has no population limit. On the other hand, real-world terrains, the distribution of natural resources and initial settlements, land use policies, and technological developments confine the actual growth of cities. Future work will include these and should give us a more realistic simulation of the evolution of cities.

## Methods

### Simulation

We implement the simulations by rasterising the entire *L* × *L* space into *L* ^2^ lattices. At each time step, a new node is generated in a place according its attraction (i.e., Π(*r*, *θ*, *t*) ∝ *ρ*(*r*, *θ*, *t*) + *C*), where *ρ*(*r*, *θ*, *t*) is the local density of the AP at location (*r*, *θ*) and time t (thus in the simulations it is the total number of nodes in a certain unit area at location (*r*, *θ*) and time *t*). and *C* is the attraction of free space for exploration. When *L* is very large, the lattice is a good approximation of continuous space (the lattice setting is supplied for the simulations). Using simulation, we prove that *r*_0_ does not affect the exponents (see Supplementary Note 1 and Supplementary Fig. 3). After obtaining the active community node distribution, we generate the road network by Voronoi tessellation around the nodes, and we calculate the corresponding socioeconomic output according to Eq. (3). We also address cases in which there are more initial seed nodes and a new seed node added after a certain number of time steps and find that our model and our results are robust (see Supplementary Note 8 and Supplementary Figs. 15–19).

### Data and pre-processing

All data sets used in this analysis are publicly available. We obtain the working and residential populations of London from the 2011 Census available at http://data.gov.uk/. The population data have an output area (OA) resolution, which is the lowest level of geography produced across all Census topics. For better visualisation, in Fig. 1d a node corresponds to a middle layer super output area (MSOA), which is ~25 times the OA population. Each MSOA possesses a population of ~5000. There are ~1500 nodes in Fig. 1d. We obtain the employed population and residential population data for Beijing from the Beijing Municipal Bureau of Statistics. The data set is provided at a *jiedao* resolution, which is the Chinese term for a statistical unit between a district and block (see the details of the refining process in Supplementary Note 4 and Supplementary Fig. 9). The residential population data are drawn from the 6th census conducted in 2010. The employed population data are from 2008. All the population data are calibrated by an increase that makes them compatible with the real situation in 2012. The AP reflects a mixture of work time and leisure time. If a workday is 8 h, the international convention for the length of a workday, the average ratio of work to leisure time for one week is ~8 × 5:(16 × 5 + 24 × 2) ≈ 1:3.

We obtain the data for road networks from https://mapzen.com/metro-extracts (the maps extracted from the Open Street Map (OSM) http://www.openstreetmap.org/). For a higher resolution, we calculate the length of all the straight segments rather than the roads, which may be composed of several segments. In the calculation of the cumulative distribution in Fig. 3, when the centre of a road segment falls within a circle of radius *r*, then the length of this segment will be accumulated.

We obtain the visible infrared imaging radiometer suite (VIIRS) nighttime light data for all the cities studied from NOAA/NGDC (http://ngdc.noaa.gov/eog/viirs/download_monthly.html). We extract the nighttime light map for each city from the global map for October 2012 and then reproject them by azimuthal equidistant projection, which is suitable for a single city. Then we rasterise the raw bitmap at a 100-m resolution (i.e., the pixel in the reprojected map corresponds to a square with an edge length of 100 m). We set the centre point of each city to be the centre of each respective projection, and this locates all points on the map at proportionately correct distances from the corresponding central area (see Supplementary Note 6).

### Data avalability

All data sets are publicly available, most of which are open access online as stated in Data and pre-processing, the census data sets of Beijing are also publicly available upon requests but not available online. All data sets are available from the authors upon reasonable request.

## Electronic supplementary material

Supplementary Information
