# Supplementary material for: Simple spatial scaling rules behind complex cities
Source: Nat Commun. 2017 Nov 28;8:1841. doi: 10.1038/s41467-017-01882-w (PMC5705765; doi:10.1038/s41467-017-01882-w)
Supplement: Supplementary file 1 — Supplementary Information [file 41467_2017_1882_MOESM1_ESM.pdf]

Supplementary Figures

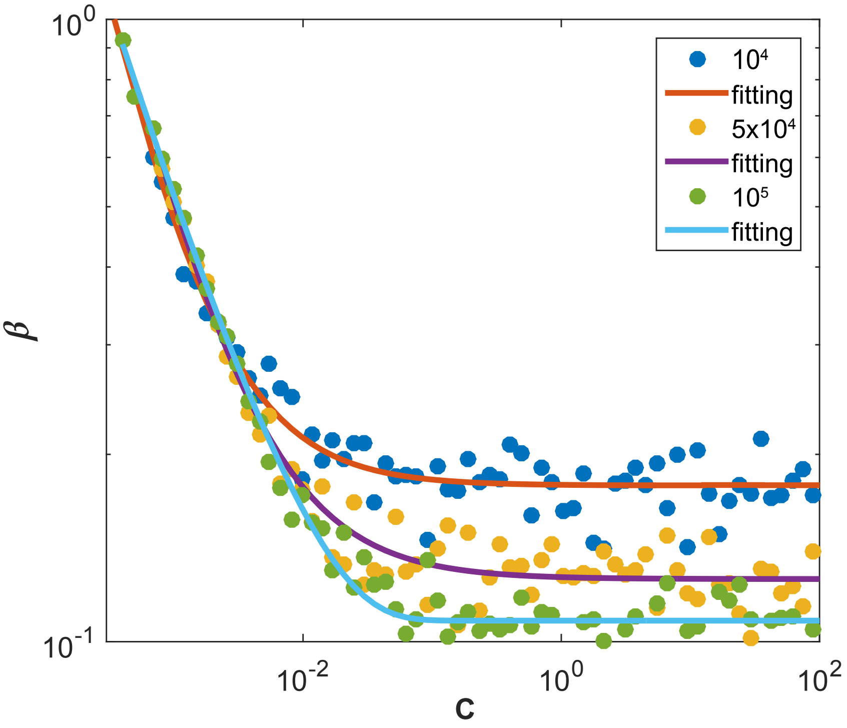

Supplementary Figure 1: The relationships between β and C with different sizes. The best fitting formula is $\beta = 0.0035C^{-0.697}e^{-46.280C}$+0.108 with $R^2 = 0.9956$ when system size is 10^5. When the size P_t is set to 5×10^4 or 10^4, the best fitting formula has similar form but the constant terms are 0.126 and 0.178, respectively. Thus, we expect that the constant term will vanish when $P_t \to \infty$.

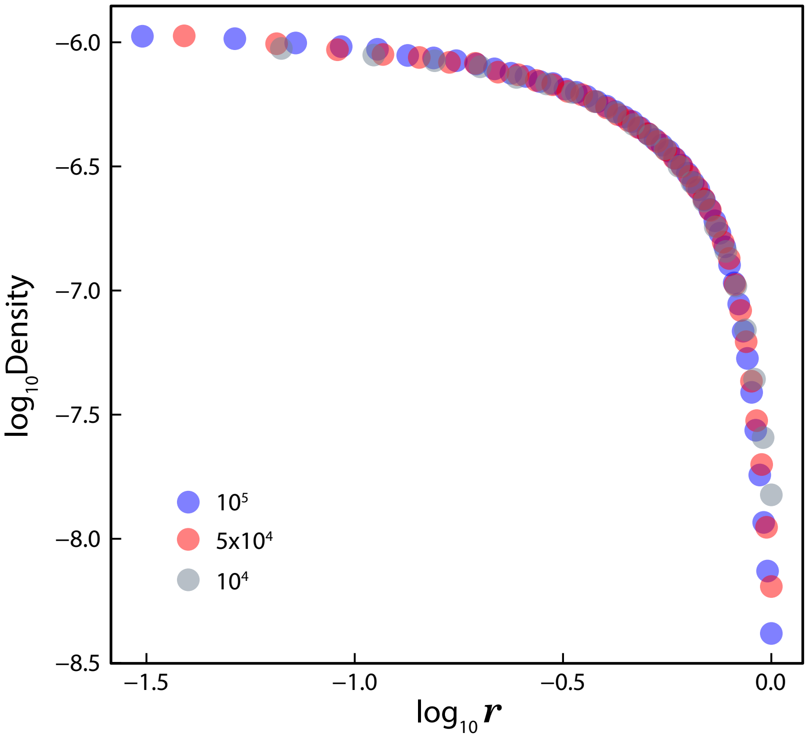

Supplementary Figure 2: Distribution of the rescaled active population (AP) density. The rescaling is performed by dividing the maximum of R_t for both x and y values. The collapse of the three lines indicates that the size effect is negligible and A in Eq. 1 is a constant. Here, $C = 1$ and different colors correspond to different system sizes.

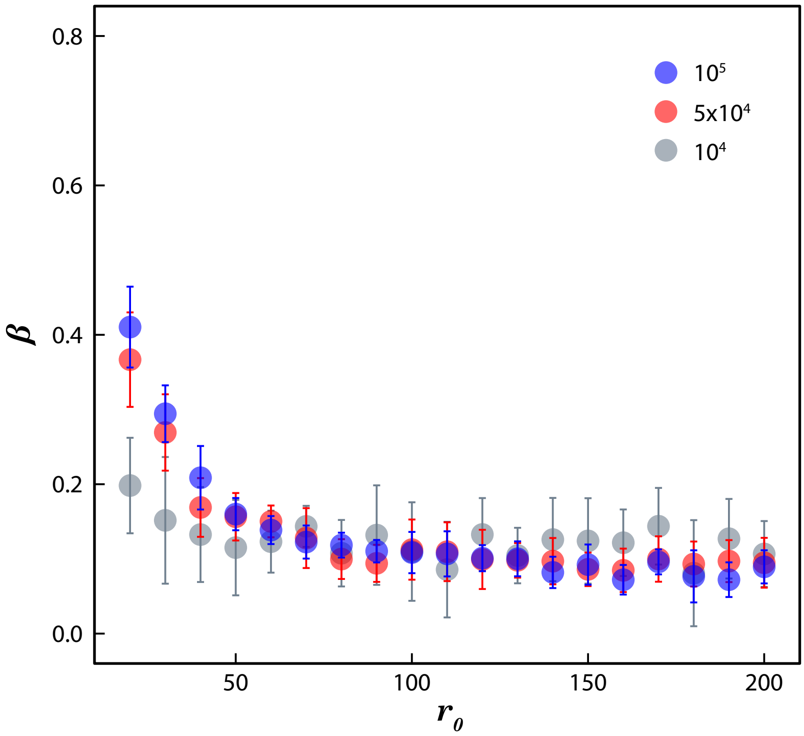

Supplementary Figure 3: β as a function of r_0. The obtained β is almost the same when r_0 is large enough, and when the system size is large enough, the fluctuation is smaller. Here, $C = 1$ and different colors correspond to different system sizes.

a

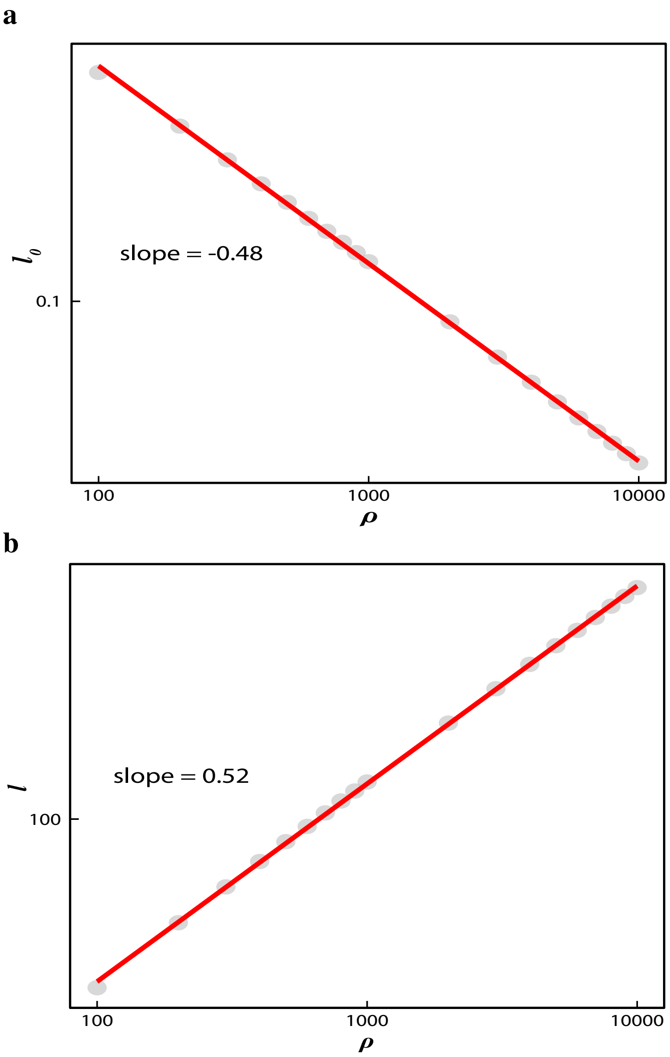

Supplementary Figure 4: Properties of the road network composed by Voronoi polygons. **a** The relationship between the average length of road segment per capita area and the corresponding AP density ρ, i.e., $l_0 \sim \rho^{-1/2}$. **b** The relationship between total road length l per capita area and AP density, i.e., $l \sim \rho^{1/2}$.

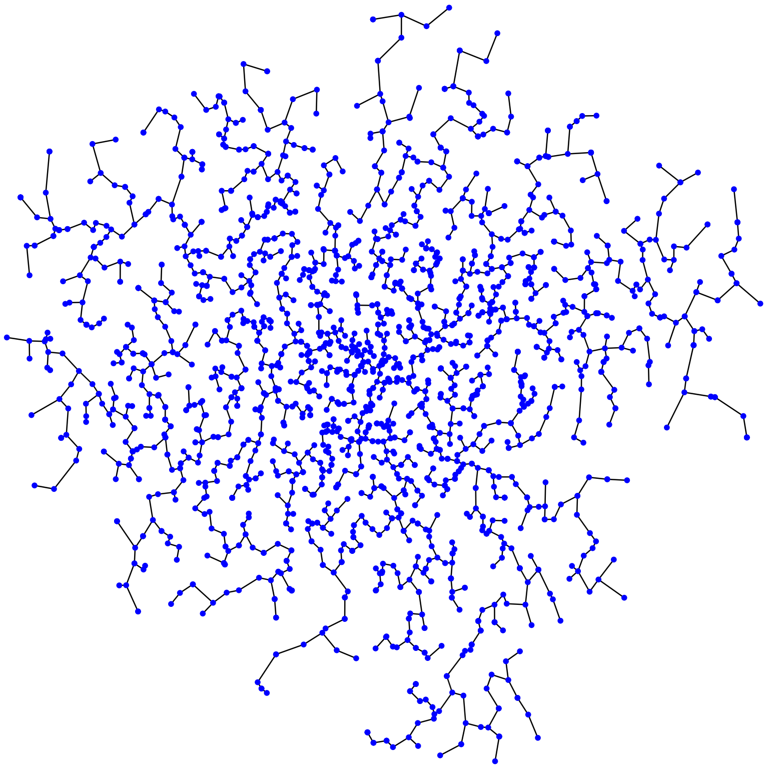

Supplementary Figure 5: A sample of road networks generated by the minimum spanning tree model.

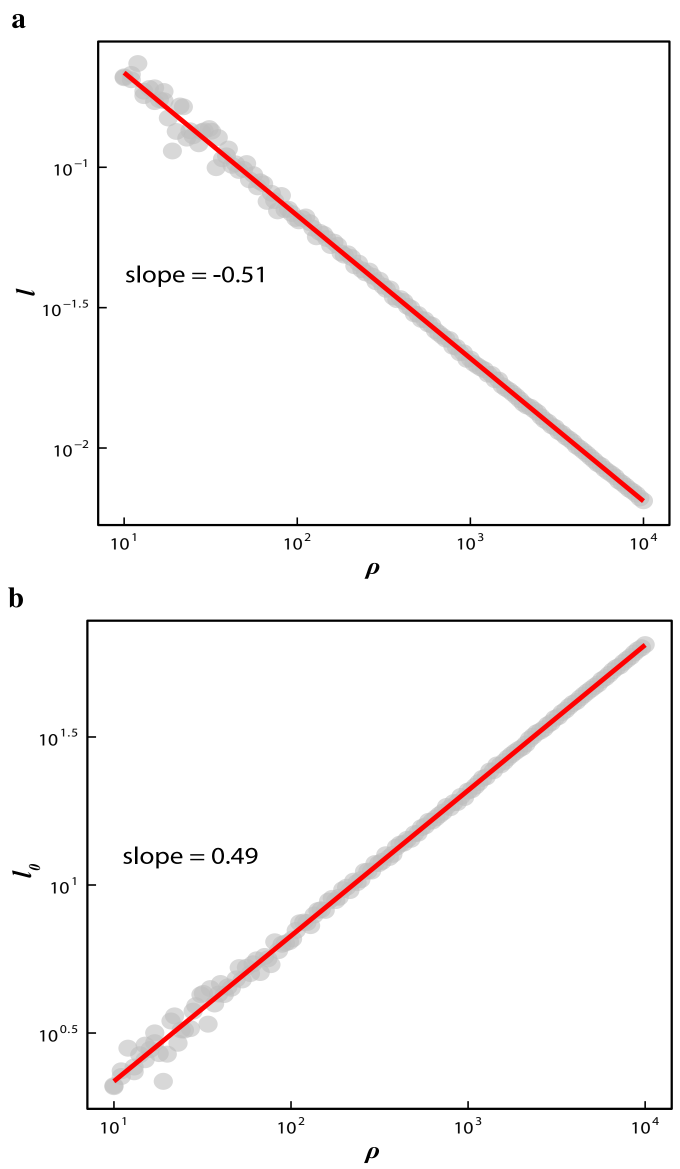

Supplementary Figure 6: Properties of the road network generated by MST. **a** The relationship between the average length of road segment per capita area and the corresponding AP density ρ, i.e., $l_0 \sim \rho^{-1/2}$. **b** The relationship between the total road length l per capita area and AP density, i.e., $l \sim \rho^{1/2}$.

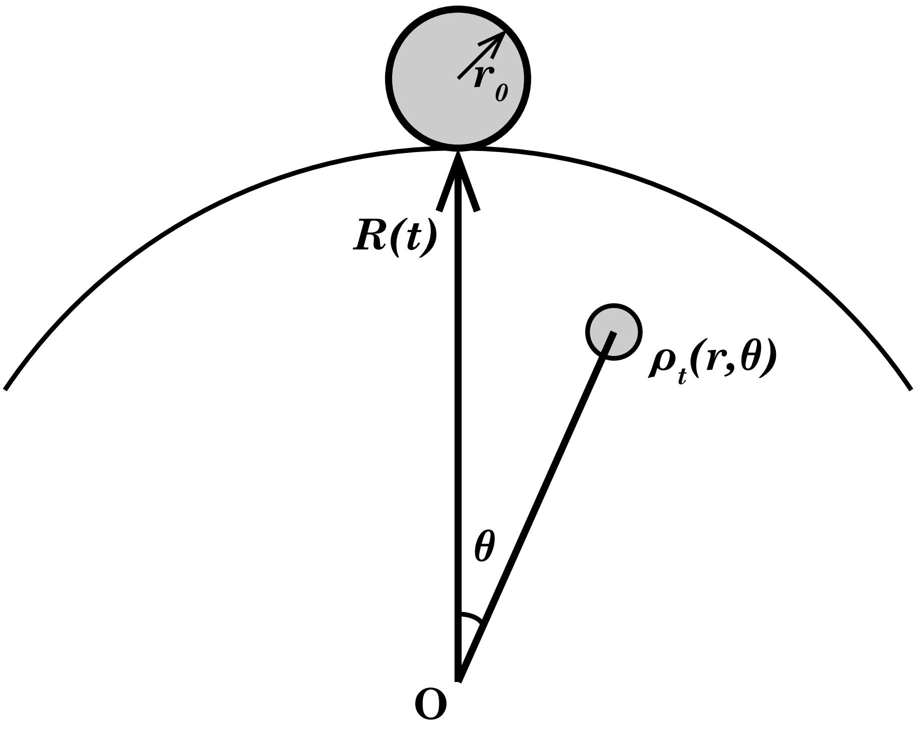

Supplementary Figure 7: Illustration of the mean field analysis in two-dimensional space.

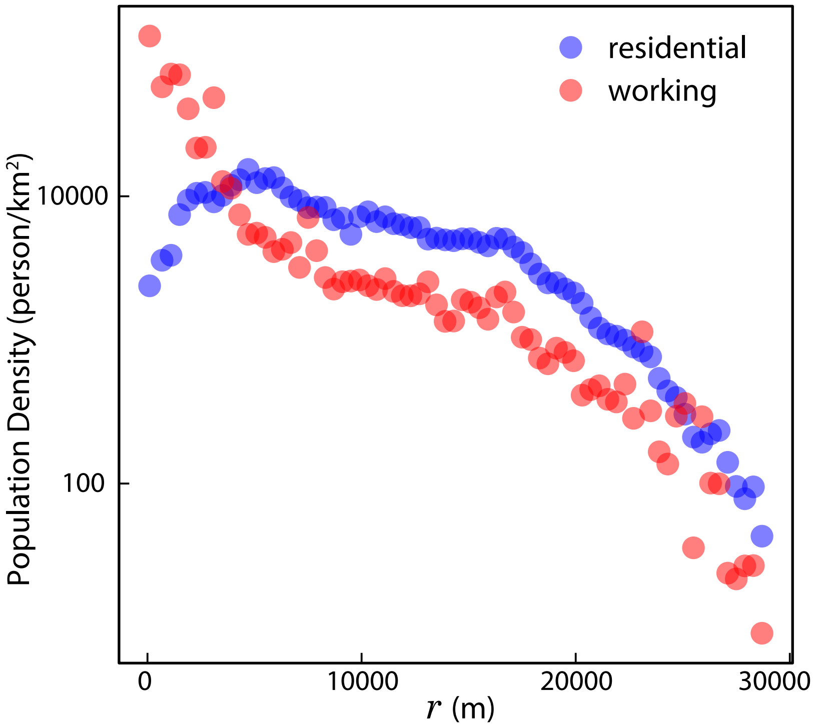

Supplementary Figure 8: The working (red) and residential population (blue) density distributions in Greater London.

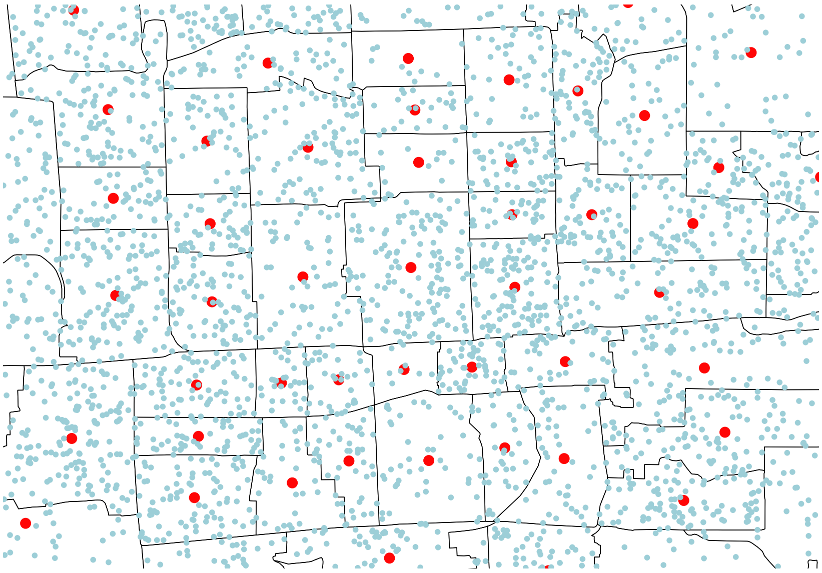

Supplementary Figure 9: The generated AP data points (blue-gray) in Beijing at *jiedao* resolution. We only have the information on total AP for each polygon (i.e., *jiedao*). To overcome the low resolution problem of the data, we generate random data points (blue-gray) within the region. The number of points is proportional to the total AP in each polygon. Each blue-gray node stands for a population of 1000, while a red node simply indicates the region, not the population size.

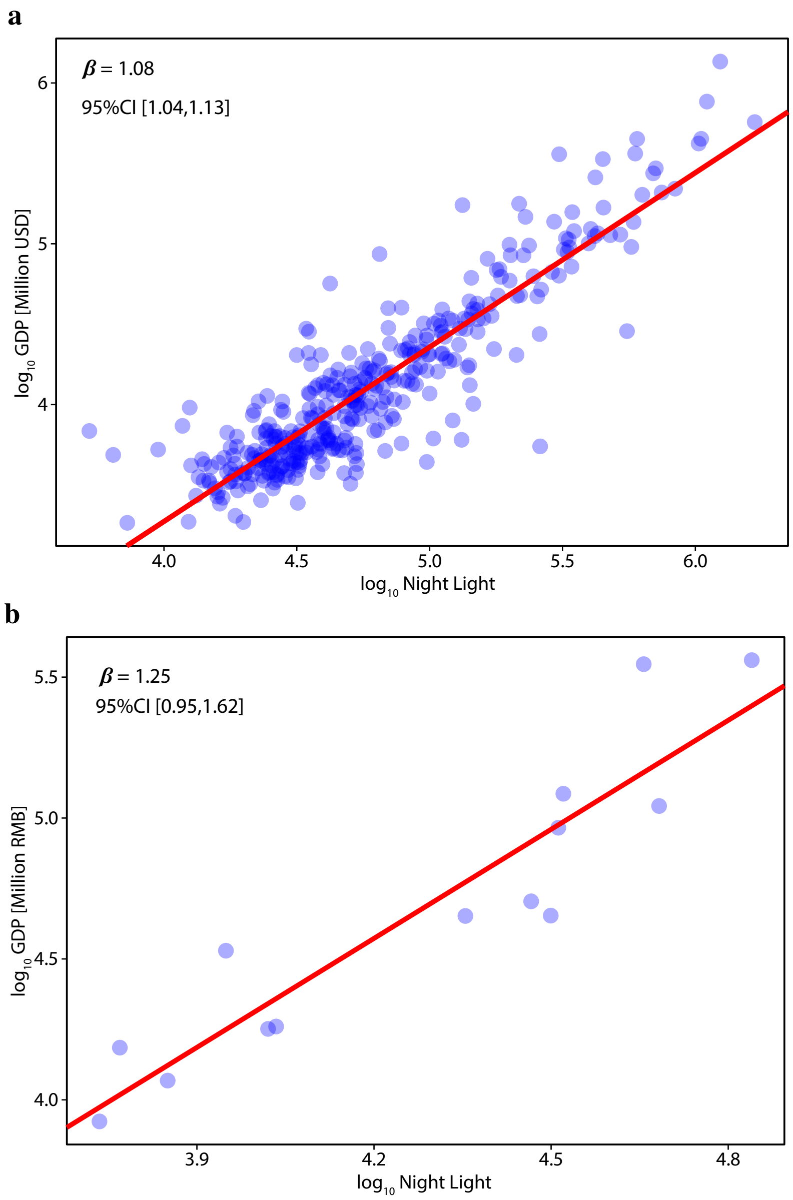

Supplementary Figure 10: The relationships between nighttime light and regional GDP at **a** U.S. metropolitan statistical area (MSA) level and **b** Beijing district level. Due to limitations in measurement methods in many countries and limited accessibility to the data, we only test Beijing as a case study. We obtain the regional GDP data from `http://www.bea.gov/` (MSAs data) and the Beijing Municipal Bureau of Statistics (at a district level). And it's worth noting that compared to the data used in Ref. [1–3], the nighttime light data in this paper is of higher resolution for both spatial and luminosity (the spatial resolution has been improved to 500m from previous 1km (`http://ngdc.noaa.gov/eog/viirs/`), and the upper limit of luminosity value is much higher than previous data which improved from 64 to 256, which now has far less over-saturation problem.

a

b

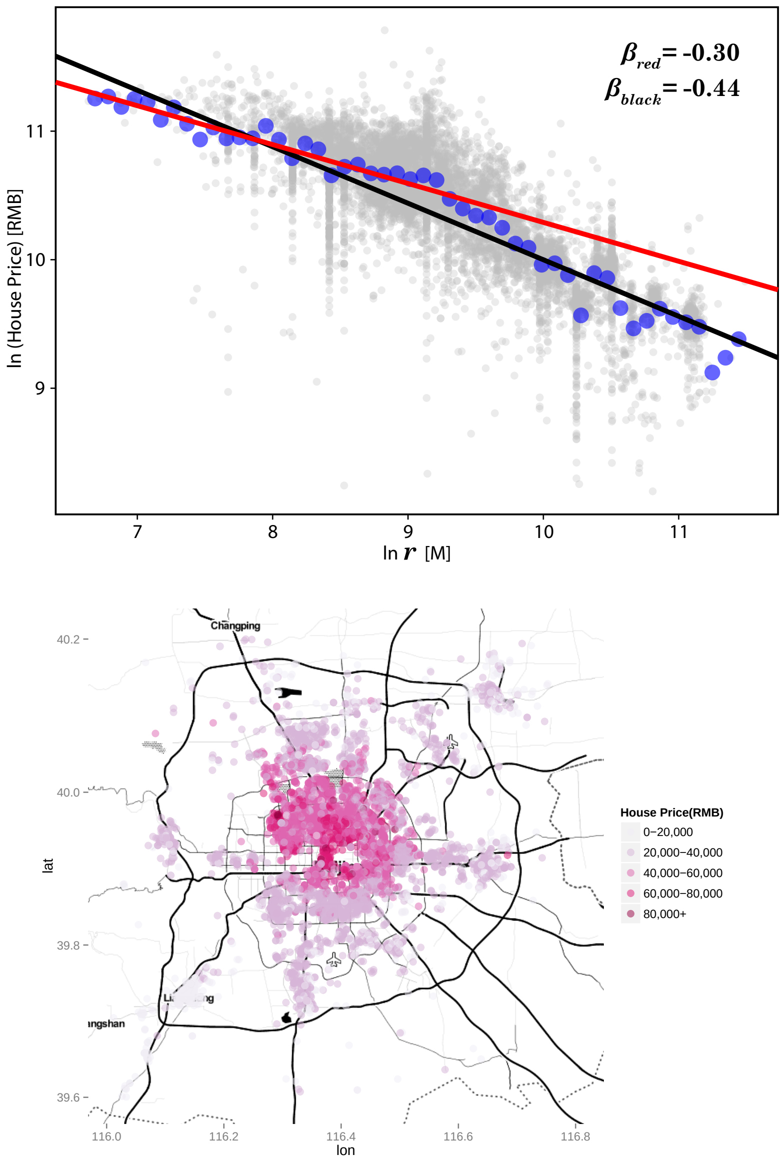

Supplementary Figure 11: **a** The house price decay in Beijing. The data are obtained from the website `www.anjuke.com` in 2013. The gray scatters are the raw data, the blue points are the average price, and the lines are obtained by OLS for different fitting regions (red for a radius of 10km, black for 15km). **b** The spatial distribution of house prices in Beijing.

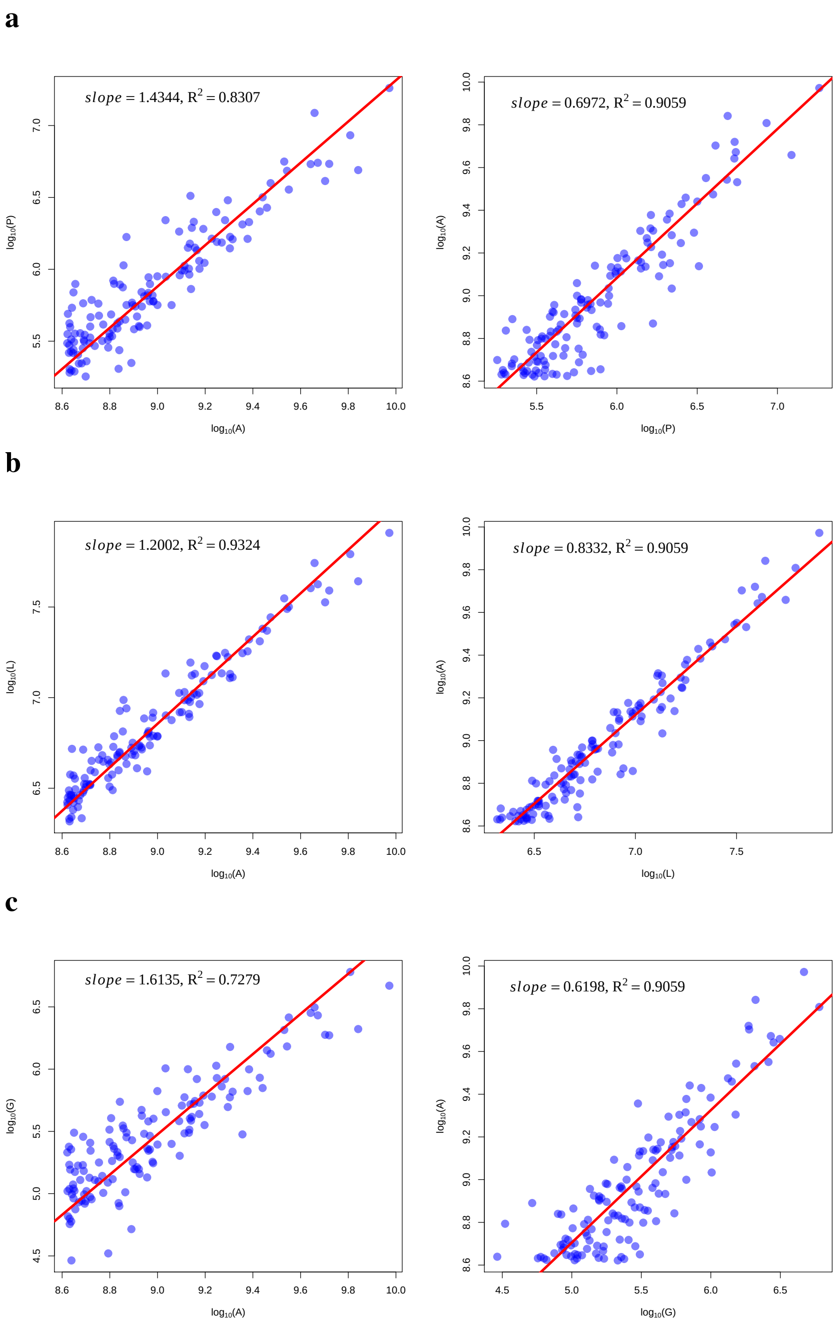

Supplementary Figure 12: Regressions by support vector machine, of which the exponents are consistent. **a** Population versus area. **b** Road length versus area. **c** Socioeconomic output versus area.

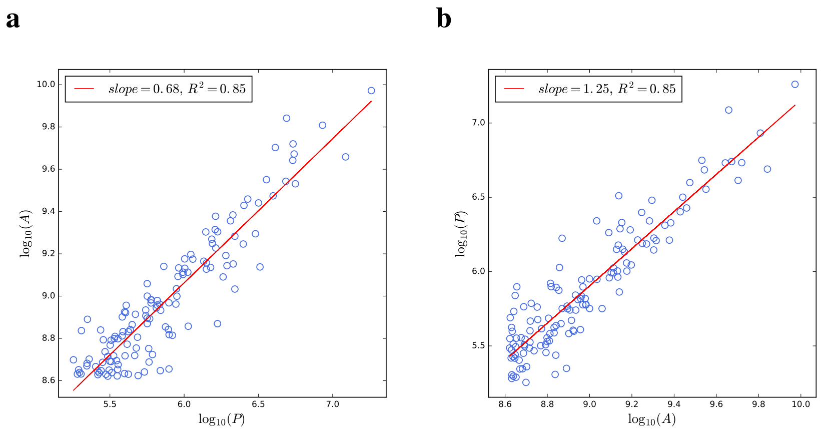

Supplementary Figure 13: Regressions by OLS, of which the exponents are not consistent. **a** Area versus population. **b** Population versus area.

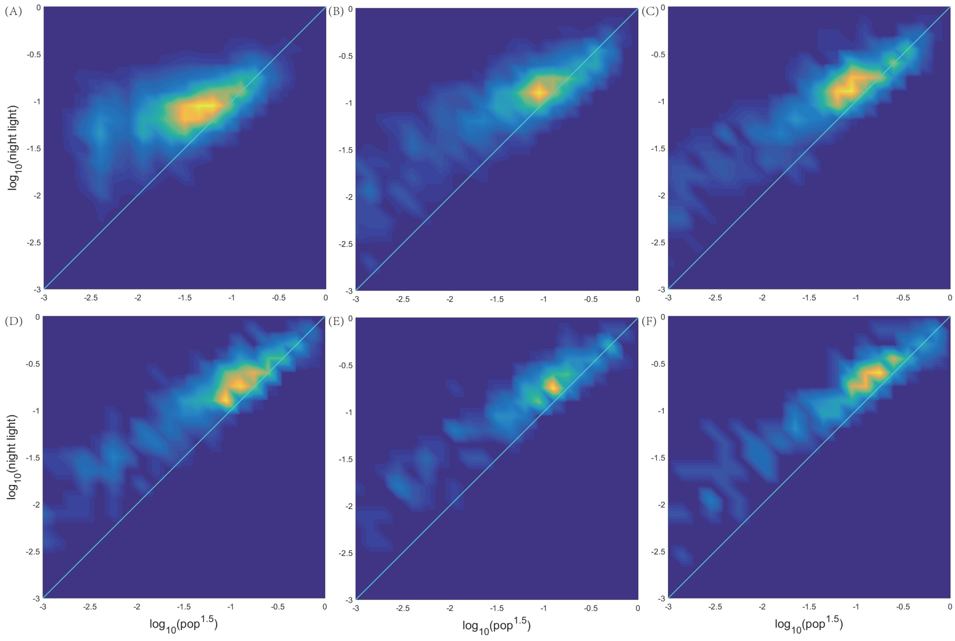

Supplementary Figure 14: Colour map of the scatter plot for correlation between nighttime light and AP 1.5 power, with different spatial resolution **a** 500m, **b** 1km, **c** 1.5km, **d** 2km, **e** 2.5km, **f** 3km. Brighter areas indicate that more data fall in that small region.

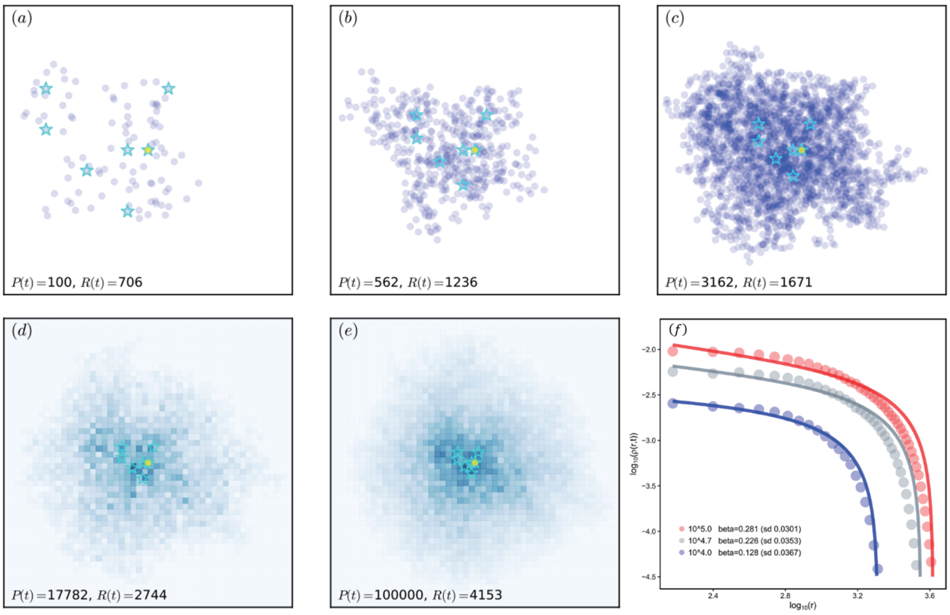

Supplementary Figure 15: The situation with 7 seed nodes. (a-e) represent the evolution of one realization at different time steps, (f) the AP density distribution. All the seed nodes (indicated as a cyan star, the (0,0) position is indicated as the yellow dot) are randomly generated within the square range of $12r_0$ with center at (0,0) (i.e., the yellow dot). Since in our model, for a new node, it can only survive if it's within a range of r_0 to any existing nodes, so we use r_0 as the basis of evaluating how far the seed nodes are away from each other. And in all our simulations as well as in the main text, $r_0 = 100$. General settings for Supplementary Figs. 15-19: (i) The node density distribution in sub-fig **f** is the average of 10 realizations. The illustrative sub-figures **a-e** represents the case of one realization. (ii) The parameters for all the simulations are the same with settings in Fig.2b with C=0.002. (iii) To increase visibility we avoid plotting the road network, which would require a higher resolution to make all the roads clearly visible (in the current figure size, the roads are covered by the nodes in the figures). (iv) When the number of nodes exceeds 10,000, we use a meshgrid normalized density plot instead of a scatter plot to more clearly show the node density distribution. In the meshgrid plots, we raster the space into 50*50 lattices, and within each lattice we count the number of nodes and normalize the lattice with the largest value. We apply settings (i)-(iv) to the remaining simulations, and avoid redundant descriptions for the remaining figures.

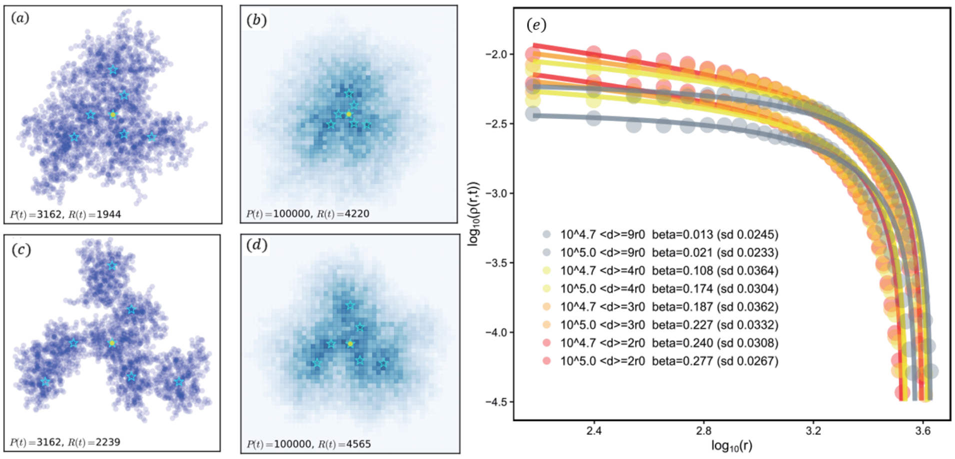

Supplementary Figure 16: The situation with fixed seed nodes formation. (a-e) represent the evolution of one realization at different time steps, (f) the AP density distribution. Each seed node is $\langle d \rangle$ away from the closest seed node. We show the simulation for the case with $\langle d \rangle$=4r_0 (a, b) and $\langle d \rangle$=9r_0 (c, d), and in **e** we present all the results with $\langle d \rangle$=2r_0 (red dots), 3r_0 (orange dots), 4r_0 (yellow dots) and 9r_0 (grey dots).

Supplementary Table

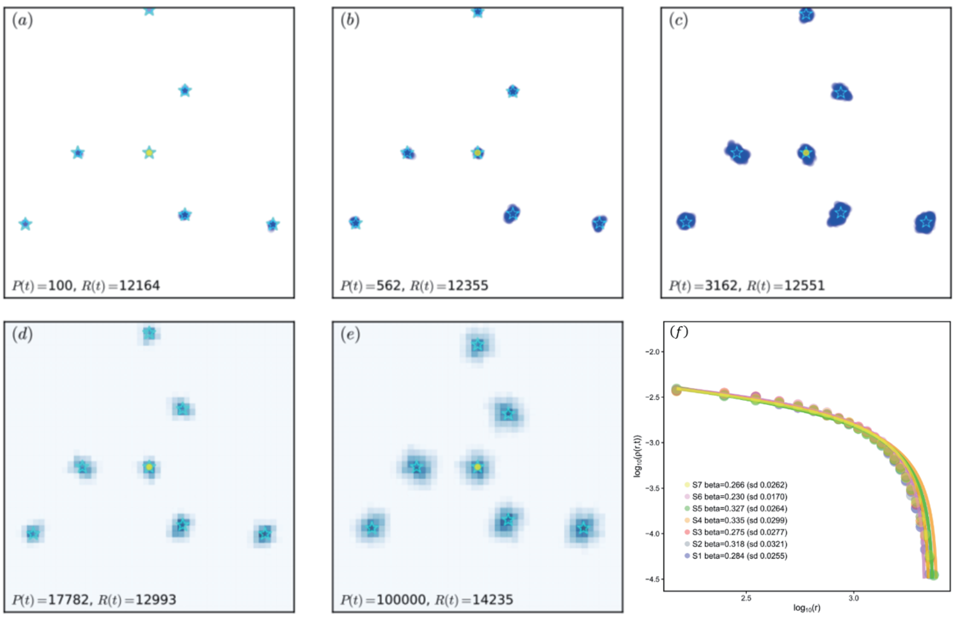

Supplementary Figure 17: The situation with 7 seed nodes far away from each other. (a-e) represent the evolution of one realization at different time steps, (f) the AP density distribution. The distance between any two closest seed nodes is $30r_0$. The size of each group varies from 11k to 16k.

Supplementary Table 1: More detailed information on cities studied in the main text

City	Country	Center	(Lat,Lon)	Radius
Amsterdam	Netherlands	Nieuwmarkt	(52.373,4.900)	5km
Beijing	China	Tian'anmen Square	(39.903,115.392)	10km
Berlin	Germany	Alexanderplatz	(52.521,13.412)	10km
Budapest	Hungary	Elisabeth Bridge	(47.493,19.051)	10km
Lille	France	Palace of Fine Arts	(50.631,3.065)	5km
London	UK	Charing Cross	(51.507,-0.128)	10km
Los Angeles	USA	City Hall	(34.052,-118.244)	15km
Milan	Italy	Duomo	(45.464,9.190)	5km
Prague	Czech	Miru	(50.075,14.436)	10km
Tokyo	Japan	Train Terminal	(35.685,139.755)	15km

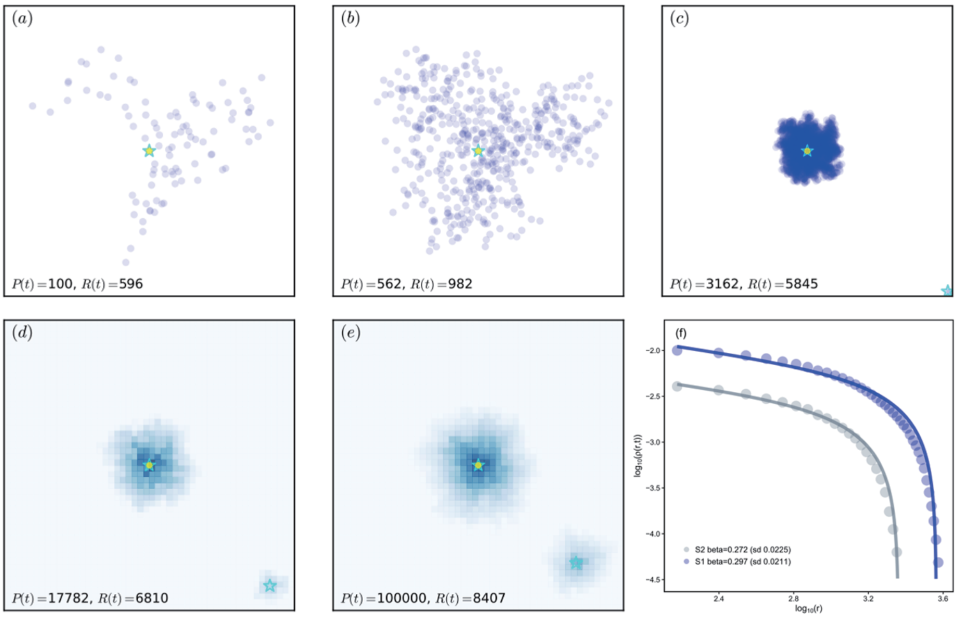

Supplementary Figure 18: The situation with adding a new seed node after the P(t) exceeds 3000. (a-e) represent the evolution of one realization at different time steps, (f) the AP density distribution. And the new seed node at the southeast of and $80r_0$ away from the initial seed node.

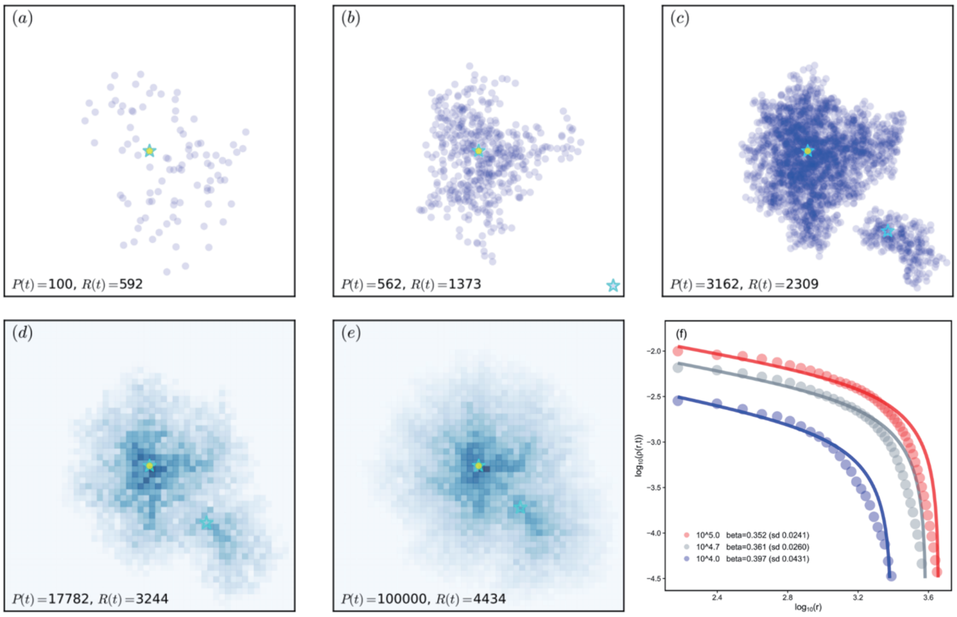

Supplementary Figure 19: The situation with adding a new seed node after the P(t) exceeds 500. (a-e) represent the evolution of one realization at different time steps, (f) the AP density distribution. And the new seed node at the southeast of and $18r_0$ away from the initial seed node.

Supplementary Notes

Supplementary Note 1: Simulations of AP distribution

In the main text our model is continuous because $\rho(r, \theta, t)$ is a continuous field. However in the simulations we must rasterize the $L \times L$ world into L^2 discrete lattices (i.e., the side length is 1) in order to calculate the value of ρ and to account for the effect of C. Thus we must use techniques to fulfill the continuity requirement.

Simulation experiments in the main text are implemented as follows. In an $L \times L$ 2-D Euclidean space the city grows by sequentially adding nodes (active communities) based on the "spatial attraction" (SA) mechanism. Specifically, when the seed node is initially located at the center of the free space, at each time step one node is generated at a random position according to probability $\Pi(r, \theta, t) \propto \rho(r, \theta, t) + C(r, \theta, t)$, where $\rho(r, \theta, t)$, at location (r, θ) and time t, is the density of the AP within the unit area (i.e., a lattice). $C(r, \theta, t)$ is a free parameter characterising the attraction of the natural endowment, which can also simulate the human preference for "empty" space. For simplicity we assume that C does not change over time and not vary across different locations. The density $\rho(r, \theta, t)$ of a lattice quantifies the number of its nodes (i.e., active communities). A new node survives only if it is sufficiently close to other existing nodes (the Euclidean distance between the new node and any other existing node must be smaller than a given threshold r_0); otherwise, it will be removed. Previously existing city nodes survive in perpetuity.

Ideally we need to set L large enough to ensure that r_0 is sufficiently large to meet the continuity assumption. In most simulations we set $L = 10^8,\ r_0 = 100$. However, since most nodes generated in this way do not survive, the simulation time will be very long. To accelerate the process, we generate nodes within the possible survival regions (approximately a square with edge length $2R_t$ whose center locats at the position of seed node).

The numerical calculation of $\rho(r, t)$

We numerically calculate the AP density decay $\rho(r, t)$ (abbreviated $\rho(r)$) by summing all the nodes within the ring with a radius from $r - \Delta r$ to $r + \Delta r$ and dividing by the area of the ring, where Δr is theoretically assumed to approach 0. To increase its practicality we set $\Delta r = r_0$ in the simulations because when Δr is too small it causes strong fluctuations and violates the continuity assumption of our model.

Note that in the simulations we separate $[0, R_t]$ into several discrete and disconnected intervals of width r_w and calculate only $\rho(\frac{n}{2} r_w),\ n = 1, 2, ..., \lfloor R_t / r_w \rfloor$ to estimate $\rho(r)$. We find that when r_w is less than r_0 the results become highly inaccurate because of violation of the continuity assumption.

The explicit form of $\rho(r,t)$

By fitting extensive simulation results, we find that the density decay formula is

$$\rho(r,t) = Ar^{-\beta}(R_t^{1+\beta} - r^{1+\beta}), \quad (1)$$

where A is a constant and R_t is the radius of the city approximated using the average greatest distance from the city center to its bounding box in the simulation.

We rescale Eq. (1) by dividing R_t, which leads to

$$\rho'(r,t) = \rho(r,t)/R_t = A(r/R_t)^{-\beta}(1 - (r/R_t)^{1+\beta}) = Ak^{-\beta}(1 - k^{1+\beta}), \quad (2)$$

where k denotes r/R_t. This shows that the rescaled formula Eq. (2) is size independent. Our simulation results are in good agreement with this rescaled form (see Supplementary Fig. 2). Figure 2b in the main text shows that the size of system (P_t) influences the absolute density magnitude but not the shape of the curves.

The explicit form of $\beta(C)$

Figure 2**a** in the main text shows that β can be estimated by a power function with an exponential cut-off of C. By using nonlinear fitting, we obtain an explicit form $\beta = 0.0035C^{-0.697}e^{-46.280C} + 0.108$, and the degree of fit is $R^2 = 0.996$ when the system size is 10^5 (see Supplementary Fig. 1). The constant term 0.108 (0.126, 0.178 for system size of 5×10^4, 10^4, respectively) would approach 0 asymptotically when the size of the system approaches infinity, which is indicated in Fig. 2**a** in the main text and in Supplementary Fig. 1.

The effect of interaction range r_0

Based on extensive simulations, we find that when r_0 is sufficiently large the estimated β is nearly constant (see Supplementary Fig. 3). As system size increases, the effect of r_0 becomes weaker (see Supplementary Fig. 3). In our simulations we set $r_0 = 100$, which is sufficiently large.

Supplementary Note 2: Simulation of road networks

Voronoi tessellation

Much effort has been dedicated to generating realistic road networks [4–6]. Some prototypical features of real road networks have been observed, such as $E = 1.5P$ (where E is the number of roads and P the number of crossroads) and $l_0 \sim \rho^{-1/2}$ (where l_0 is the average length of road segments within a unit square and ρ is the corresponding AP density at that location) [4].

For simplicity, in our model we employ the standard Voronoi tessellation based on the AP distribution generated by our model [7,8] (see Fig. 1bc in the main text) to simulate the road network and capture its significant statistical features.

Our simulations show that $l_0 \sim \rho^{-1/2}$ and $l \sim \rho^{1/2}$ are reproduced in the Voronoi tessellation (see Supplementary Fig. 4). In some simple situations there is solid evidence for both relationships. For example, in a square lattice the total length of polygons generated within a capita area are also perfect lattices. Here $l_0 \sim (S/N)^{1/2} \sim \rho^{-1/2}$ and $l \sim 2\rho l_0 \sim \rho^{1/2}$, where S is the total area and N is the number of nodes.

In addition, we can also prove that the number of intersections in the road network of our model is proportional to the number of nodes (i.e., active communities). If we define P to be the number of intersections, E the number of roads, and P_t the number of active community (i.e., the number of faces if we treat the voronoi graph as a polyhedron), according to the Euler formula we obtain $(1+P) - E + P_t = 2$, where 1 is the virtual node (i.e., an intersection) at an infinitesimal distance. When $\deg(P) \geq 3$ and $\sum \deg(P) = 2E$, then $E \geq \frac{3}{2}(P+1)$, the lower boundary of which is maintained in the usual random scenario consistent with empirical findings. We thus obtain $\frac{3}{2}(P+1) \leq E \leq 3P_t - 6$ and $P \leq 2P_t - 5$. Moreover, Voronoi tessellation can also guarantee fairness for two nodes near a road, i.e., the distances required for each of them to access the road are the same [7].

Minimum spanning tree

To verify that the details of the road network generating procedure do not affect its statistical features, we test the basic equations $l_0 \sim \rho^{-1/2}$ and $l \sim \rho^{1/2}$ for the simulated road networks generated by a minimum spanning tree (MST) (see Supplementary Fig. 5). In an MST model, when a node is added to the city a new link is added to connect the new node with its nearest neighbour (if there are several nearest neighbours at the same distance, then one of them is randomly selected for connection). Our simulation results indicate that the equations are also validated by MST (see Supplementary Fig. 6).

Note that $l_0 \sim \rho^{-1/2}$ holds for MSTs because when the considered regions are small ρ nodes approximately evenly disperse in them. Then the average distance between two nearest nodes is $l_0 = 1/\sqrt{\rho}$, and the total length of the roads is $l = \rho l_0 = \rho^{1/2}$.

Supplementary Note 3: The Mean-Field analytical results of the model when $C \to \infty$

According to the SA mechanism introduced in the main text we can simulate the growth of a city, but instead of merely reporting the numeric results we asymptotically derive analytical results for the scaling exponents (namely let $t, L \to \infty$) for a special case when $C \to \infty$ by an Mean-Field (MF) theory based on growth rather than static assumptions.

The shape of the city is irregular and anisotropic when the simulation time step t is smal; but it will shift to a symmetric system with a rough perimeter when t is

large. Under the MF approximation, the radius of the whole system, denoted R_t, grows linearly with time t

$$R_t \sim t, \tag{3}$$

where R_t is the longest distance between the city center and the system bounding box.

When $C \to \infty$, each place in the 2-D space has an equivalent probability (i.e., we can assume that $\Pi(r, \theta) = 1/L^2$) of accepting a newly generated node at each time step. Thus the probability that a new node will locate at the boundary of the system (see Supplementary Fig. 7) is proportional to its perimeter ($2\pi R_t$) and independent of time. The average time span between two nodes being added at the perimeter of the system is then proportional to $1/R_t$. To increase the radius of the system by one unit, we need a large number of nodes (the number of nodes increases with $\sim R_t$) to fill out the perimeter. Thus the average time required for the radius of the system to increase by one unit is nearly constant ($\sim R_t \cdot (1/R_t)$). This means that the radius grows at a constant speed, which yields Eq. (3).

According to Eq. (3) the total area of the system increases in time squared

$$A_t \sim R_t^2 \sim t^2. \tag{4}$$

To derive the total number of nodes P_t in the system, we calculate the node density $\rho(r, \theta, t)$ at any spatial location with polar coordinate (r, θ) and time t

$$\rho(r, \theta, t) = \int_{\tau_r}^{t} \frac{1}{L^2} ds \sim (t - \tau_r) \sim (R_t - r), \tag{5}$$

where ds is the infinitesimal time and τ_r is the time when the system radius is r. Because the probability at which the infinitesimal area $d\sigma$ accepts a new node is a constant ($1/L^2$), the average density of the AP in this infinitesimal area is the accumulation of nodes born between time steps τ_r and t. We set $\beta = 0$ in Eq. (1) and derive Eq. (5), indicating that Eq. (1) is a general form. The total population P_t can be computed by integrating Eq. (5),

$$\begin{aligned} P_t &= \int_0^{R_t} \int_0^{2\pi} \rho(r, \theta, t) r dr d\theta \\ &= 2\pi \int_0^{R_t} r^{1-\beta} R_t^{1+\beta} - r^2 dr \\ &= 2\pi \left(\frac{R_t^3}{2-\beta} - \frac{R_t^3}{3} \right) \sim R_t^3. \end{aligned} \tag{6}$$

According to Eqs. (6) and (4) we obtain the scaling relationship between area and population $A_t \sim P_t^{2/3}$.

We next analyse the total road length and assume that the road length per capita is $l(r, \theta, t)$ at time t. According to $l \sim \rho^{1/2}$ the total road length of the entire

network is

$$
\begin{aligned}
L_t = \int l_t d\sigma &\sim \int_0^{R_t} \int_0^{2\pi} l(r,\theta,t) r dr d\theta \\
&= \int_0^{R_t} \int_0^{2\pi} \rho(r,\theta,t)^{1/2} r dr d\theta \sim R_t^{5/2}.
\end{aligned} \tag{7}
$$

Combining Eqs. (6) and (7) we obtain the scaling relationship $L_t \sim P_t^{5/6}$.

We also assume that the total local interactions per capita are equal to $g(r, \theta, t)$, which is proportional to the local AP density times the local road volume, assuming that all local interactions occur along the road. Thus $g(r, \theta, t) = \rho(r, \theta, t) l(r, \theta, t) \sim \rho_t^{3/2}$. The total number of socioeconomic interactions in the system is then

$$
\begin{aligned}
G_t = \int g_t d\sigma &\sim \int_0^{R_t} \int_0^{2\pi} g(r,\theta,t) r dr d\theta \\
&= \int_0^{R_t} \int_0^{2\pi} \rho(r,\theta,t)^{3/2} r dr d\theta \sim R_t^{7/2}.
\end{aligned} \tag{8}
$$

Hence G_t and P_t have the scaling relationship $G_t \sim P_t^{7/6}$. Because the total output in the system is proportional to the total number of interactions, the socioeconomic interactions in the system scale with the population at power 7/6.

Empirical validation

To test whether the relations

$$P_t \sim R_t^3, \;\; G_t \sim R_t^{7/2}, \;\; L_t \sim R_t^{5/2}$$

hold in a real-world scenario, we use data from US cities. We first obtain shapefiles of all the cities on the US Census website (`https://www.census.gov/geo/maps-data/`) in which the boundary is closer to the metropolitan statistical area rather than to the administrative boundary. We choose the top 150 cities and calculate the total area A of each. We then download all the road networks in US from the USGS website (`https://www.usgs.gov/`) and calculate the total road length L within urban areas. There are 19,371,674 roads in US, approximately one third of which (6,732,730) are in urban areas. Finally we obtain the night light data from NOAA/NGDC (the same source as in the main text), and calculate the total luminosity G of each city.

Because the shape of real-world cities is usually irregular (e.g., they are not round or square) it is difficult to calculate their radius. Instead we calculate their effective radius $\sqrt{A/\pi}$ and test all the scaling exponents on area A. We then multipy by 2 to get the scaling exponents on R_t. Supplementary Fig. S12 shows that the exponent of population P on area A is 1.434, which yields 2.869 on radius R_t. For G it is 3.227, and for L it is 2.400.

Here we encounter a technical issue related to the ordinary least squares (OLS) assumption. Because these data do not statisfy the OLS assumption, the regression exponents are not consistent when we do a coordination transform. For example, when we do an OLS regression on the population it gives us $A \sim P^{0.68}$ (see Supplementary Fig. 13**a**); if the method were consistent it would give us $P \sim A^{1/0.68} = A^{1.47}$, but when we do an OLS on an area it gives us $P \sim A^{1.25}$ (see Supplementary Fig. 13**b**), which is far less than 1.47. The reason partially lies in the nature of the data and partially in the OLS process, which minimizes the RSS distance of points to the line and causes inconsistency in the coordinate transform. Thus to maintain consistency we employ a support vector machine for regression, and this gives us consistent results (see Supplementary Fig. S12).

Supplementary Note 4: Population distributions of London and Beijing

London

The main text indicates that the AP distribution follows a power law. Supplementary Fig. 8)) shows that the difference between working and residential population densities distribution is significant, and none of them are power law. In central areas that are often non-livable or too expensive the residential population density is low but the working population density high.

Beijing

The population data for Beijing are at a *jiedao* resolution, which is a lower resolution than the London data. Thus we perform some pre-processes to increase the resolution. There are fewer than 200 original data points within our research area. Each red point denotes the region (the polygon in Supplementary Fig. 9) but not the population size. Thus we generate AP nodes randomly at each *jiedao* (see Supplementary Fig. 9). Each generated point (light gray) represents 1000 persons, and the number of points is proportional to the total population within this region. If we had more information on the building situation in each area (e.g., land use, proportion of built-up area, and building density), we would be able to obtain a more realistic distribution.

Supplementary Note 5: More details for empirical studies of real cities

The high-quality working population data used in this paper are unfortunately usually inaccessible. We were only able to acquire this data for London and Beijing. These data are also usually affected by such technical problems as measurement techniques. In contrast, road network and nighttime light data are widely available, especially for large cities, and using these we analyze 10 large representative cities worldwide (see Table 2 in the main text and Supplementary Table 1).

Fitting regions for spatial scalings

To study the spatial scaling of real cities, we need to determine the range of distance to fit the data and estimate the exponent. We determine the lower and upper cut-offs of the distance such that the intermediate region remains. This allows us to ignore the influences of noise and non-linear effects [9–11]. We set the lower cut-off of the distance to the city center at 500 meters. The upper cutoff is calculated by the reported urban area of the city. In general this is the radius of the fully urbanized area. In Supplementary Table 1 we provide detailed information on the location and name of the city center and the range of the city (in Table 2 in the main text, we only give the exponents). We cross check this information from Wikipedia, Geohack, and Google Earth.

In addition, the U.S. Census has a good quality, but it only counts the number of residents in a certain area as residential population and the number of residents who have a job as working population (the resident may work in other places, but they don't record this information). So the working population of a place from U.S. Census is the population who have jobs and living that area, rather than the working population who are really working in a certain area. So these two working population are totally different, the latter one is what we want but can not be obtained from U.S. Census.

Supplementary Note 6: The correlation between socioeconomic interactions and nighttime light within cities

Previous studies [1–3] indicate that nighttime light data is a good proxy for GDP (a typical socioeconomic output quantity) at nation or state level, and nighttime light data has been widely used in GDP analysis [12]. And it's natural to assume that the number of socioeconomic output is proportional to the socioeconomic interactions (see Ref. [13] and references therein, and this assumption is also partially validated in Ref. [14]), since human activities are the very reason behind all socioeconomic outputs. So the nighttime light can be a good proxy of socioeconomic interactions at such a relatively large scale.

Yet there are concerns about whether it is also suitable at a finer scale (e.g., at a city, sub-city(district) and even community level), since at a high spatial resolution (especially at the community level), the luminosity is more related to the density of roads, the installation of lights and the type of the communities (e.g., business area naturally have more lights than residencial and industrial areas where the interactions are more likely to occur during the day). Although there is a disadvantage to using nighttime light data as a proxy of interactions at such a high spatial resolution (e.g., community level), luminosity data is the best among all available open-source data for estimating human activity and interaction. Compared to the data used in Refs. [1–3], the nighttime light data that we have used has higher spatial resolution and luminosity upper limit (the spatial resolution has been improved to 500m from the previous 1km (https://ngdc.noaa.gov/eog/viirs/), and the upper

limit of the luminosity value is much higher than in previous data, which has been improved from 64 to 256, and there is a far smaller over-saturation problem.

In orde to justify the correlation between interactions and nighttime luminosity, we first test it at the city and sub-city (district) level and find that luminosity is a good indicator of socioeconomic outputs at both city and sub-city scale – the correlation between the luminosity and the regional GDP of cities is high (see Supplementary Fig. 10). As long as the assumption that the level of socioeconomic output is proportional to the socioeconomic interactions [13, 14], then the correlation between interactions and nighttime luminosity should hold at the city and sub-city level.

Although at a community level, we don't have corresponding high resolution socioeconomic output data, yet the active population concept proposed in our paper provides a means of estimating the number of interactions there, which allows us to directly test the correlation between luminosity and interactions. Although estimating the number of interactions will also be affected by land use type, a larger population usually has a higher level of interaction.

We then performed a resolution robustness test on the correlation at a finer resolution (with 500m, 1km, 1.5km, 2km, 2.5km, 3km, see Supplementary Fig. 14A-F, respectively). We find that when the population density is low, the estimation from the active population deviates from the empirical night light data, which may be because the luminosity is more related to the density of roads. Thus when the population density is low, the night light may not be that low due to the impacts of roads (see Supplementary Fig. 14A). At a larger scale the deviations decrease (see Supplementary Fig. 14A-F). The point cloud is more concentrated along the diagonal, which indicates that nighttime light is a good proxy for testing our model within cities. We find that 1km*1km is a scale that maintains both a finer spatial resolution and fewer minor deviations.

Supplementary Note 7: Rent price

We can also derive additional variables under framework of our model with reasonable assumptions. For example, following Bettencourt's work [13] we can evaluate the average rent price of a given city using its total GDP output divided by area. Then the rent price is $P_{\text{rent}} \propto P_t/A_t \propto R_t^{3/2} \propto P_t^{1/2}$. Then the scaling between the average rent price in a city and the city size exhibits an exponent $1/2$, which has been validated in the literature [13]. If we assume that the local rent price is proportional to demand and local socioeconomic development level, then this yields $p(r, \theta, t) \propto g(r, \theta, t)\rho(r, \theta, t) \propto \rho^{5/2}$, where $p(r, \theta, t)$ is the local rent price density in the location (r, θ) and time t, and $g(r, \theta, t)$ is the local interaction density. Then we derive that $p(r) \propto r^{-5/2\beta}$ in the downtown area, which is qualitatively supported by the empirical results (see Supplementary Fig. 11). However, the empirical exponent in the real data deviates a bit with our model's prediction of Beijing, whose $\beta = 0.09$, where the prediction is about -0.225, whereas the empirical exponent is -0.30 for the downtown area (with radius equals 10km) or

-0.44 for a larger region (with radius 15km). Due to the nature of rent price, there's not dramatic decay even in urban fringe as compared to population density. We suspect that some key factors that affect prices may be neglected in our simple assumption. We only get access to high quality house price data in 2013 from `www.anjuke.com` and we treat it as a proxy for rent price. Although some research indicates that rent price can be used to predict house price [15], other results indicate that the relationship is not explicit [16]. Nevertheless, we clearly observe that house prices decay from the center (see Supplementary Fig. 11).

Supplementary Note 8: Simulations of AP distribution with multiple seed nodes and a new seed node after certain time steps

The goal of our paper is to propose an analytical model that is both general and simple. The one seed node assumption is the simplest initial condition that allows us to illustrate the typical growth process of a monocentric city. Yet there might be concerns about whether the model is robust with respect to the initial conditions with multiple seed nodes.

If the seed nodes are initially close to each other in a small area, the result is the same as that of a single seed node after several time steps with results identical to those shown in Fig. 2b.

Thus we examine a case in which the distances between seed nodes are neither close nor too far away. We find that the clusters first grow separately and, after several time steps, merge together. We find that early on the spatial distribution of multiple seed nodes strongly affect the morphology of the city (the spatial distribution of seed nodes becomes the backbone of the city morphology, see Supplementary Fig. 15bc), but later in the urban evolution the impact of seed nodes fades (see Supplementary Fig. 15de). We also find that the power exponent β of the density distribution is affected by the presence of multiple seed nodes, which is a little less than the results shown in Fig. 2b (especially at early periods, see Supplementary Fig. 15f), yet the population density continues to be robust (only the β varies). In later periods the impacts weaken. And we also found that the power exponent β of density distribution is affected by the presence of multiple seed nodes which is a little bit lower than the results shown in Fig.2b (especially at early periods, see Supplementary Fig. 15f), yet the population density form is quite robust (only the β varies); and when it comes to late periods, the impacts also become weaker.

To systematically test the impact on density distribution, we carry out experiments with fixed seed-node formations in which only the distance between seed nodes varies (see Supplementary Fig. 16). We do four simulations (each distinguished by a different color) with an increasing distance between each seed node, and in each simulation we also record the node density distribution at different system sizes (i.e., when P(t)=5*10^4 and 10^5). Supplemetary Fig. 16e shows simulations in which there is a flatter distribution (indicated by a smaller β value) when the system size is 50K (i.e., $10^{4.7}$) due to the dispersed distribution and separate

development of several seed nodes. When more nodes are added to the system, the slope becomes steeper and closer to that shown in Fig. 2b (see the system size 100K). This finding holds for all four cases with different distance $\langle d \rangle$ values between the seed nodes.

When we fix the final size of the system (e.g., 10^5 nodes), the increased distance $\langle d \rangle$ between seed nodes produces a flatter distribution (see Supplementary Fig. 16**e**). When $\langle d \rangle$=9r_0 it is much flatter and no longer smooth. We also test cases with a longer distance between seed nodes. When reach a certain distance (e.g., 20-30r0 for a system with 10^5 nodes), the results become simple again, and all seed nodes eventually resemble separate towns of slightly differing sizes (see Supplementary Fig. 17). Examining all seven clusters in the system, we find the node density distributions are almost the same as those shown in Fig. 2b. Yet randomness still plays a role in different clusters and causes slight differences in the β values.

Another scenario that we may observe during urbanization is the case with a new seed node added somewhere after a certain number of time steps t_c, and we carry out experiments using different conditions.

When a new seed node is at the fringe of a city about to be urbanized, the impact of the new seed node is negligible. It does not affect other nodes at the fringe provided there are no subsequent interventions.

When a seed node is located far from a city center and does not merge with it within a certain number of time steps, and if there are no subsequent interventions, the new seed node grows and forms a small town (see Supplementary Fig. 18). Investigating the impacts of further policy impacts involves more factors and is beyond the scope of this paper and would require a more systematical study in the future.

And the most interesting scenario is still the case where the new town and the previous city can finally merge together. The new seed node (new town) expands the area of the city during the early periods of growth (blue points in Supplementary Fig. 19f, which do not decrease as quickly as those shown in Fig. 2b). During later periods in the urbanization process the impact of new towns weakens. The distribution tightens (i.e., β is smaller and more similar to β in Fig. 2b), which indicates that the form of density distribution produced by our model is more stationary.

Supplementary References

[1] Sutton, P. C., Elvidge, C. D. & Ghosh, T. Estimation of gross domestic product at sub-national scales using nighttime satellite imagery. *Int. J. Ecol. Econ. Stat.* **8**, 5–21 (2007).

[2] Chen, X. & Nordhaus, W. D. Using luminosity data as a proxy for economic statistics. *Proc. Natl Acad. Sci. USA* **108**, 8589–8594 (2011).

[3] Henderson, J. V., Storeygard, A. & Weil, D. N. Measuring economic growth from outer space. *Am. Econ. Rev.* **102**, 994–1028 (2012).

[4] Barthélemy, M. & Flammini, A. Modeling urban street patterns. *Phys. Rev. Lett.* **100**, 138702 (2008).

[5] Barthélemy, M. & Flammini, A. Co-evolution of density and topology in a simple model of city formation. *Netw. Spat. Econ.* **9**, 401–425 (2009).

[6] Strano, E., Nicosia, V., Latora, V., Porta, S. & Barthélemy, M. Elementary processes governing the evolution of road networks. *Sci. Rep.* **2**, 296 (2012).

[7] Aurenhammer, F. Voronoi diagrams—a survey of a fundamental geometric data structure. *ACM Computing Surveys (CSUR)* **23**, 345–405 (1991).

[8] Okabe, A., Boots, B., Sugihara, K. & Chiu, S. N. *Spatial tessellations: concepts and applications of Voronoi diagrams*, vol. 501 (John Wiley & Sons, 2009).

[9] Kantz, H. & Schreiber, T. *Nonlinear time series analysis*, vol. 7 (Cambridge university press, 2004).

[10] Lacasa, L. & Gómez-Gardeñes, J. Correlation dimension of complex networks. *Phys. Rev. Lett.* **110**, 168703 (2013).

[11] Eckmann, J.-P. & Ruelle, D. Ergodic theory of chaos and strange attractors. *Rev. Mod. Phys.* **57**, 617 (1985).

[12] Shi, K. *et al.* Evaluating the ability of npp-viirs nighttime light data to estimate the gross domestic product and the electric power consumption of china at multiple scales: A comparison with dmsp-ols data. *Remote Sens.* **6**, 1705–1724 (2014).

[13] Bettencourt, L. M. A. The origins of scaling in cities. *Science* **340**, 1438–1441 (2013).

[14] Schläpfer, M. *et al.* The scaling of human interactions with city size. *J. R. Soc. Interface* **11**, 20130789 (2014).

[15] Hargreaves, B. What do rents tell us about house prices? *Int. J. Housing Markets and Analysis* **1**, 7–18 (2008).

[16] Gallin, J. The long-run relationship between house prices and rents. *Real Estate Econ.* **36**, 635–658 (2008).
